# An elevated urinary albumin-to-creatinine ratio increases the risk of incident cardia-cerebrovascular disease in individuals with type 2 diabetes

**DOI:** 10.1186/s13098-024-01256-5

**Published:** 2024-01-31

**Authors:** Jie Tao, Dasen Sang, Xinxin Zhang, Xin Liu, Guodong Wang, Shuohua Chen, Shouling Wu, Wei Geng

**Affiliations:** 1 Department of Cardiology, Baoding NO. 1 Central Hospital, N0. 320, Changcheng Street, Baoding, Hebei China; 2 Department of Cardiology, Kailuan General Hospital, 57 Xinhua Road(East), Tangshan, Hebei China

**Keywords:** Cardia-cerebrovascular disease, Type 2 diabetes, Albuminuria, Urine albumin-to-creatinine ratio, Risk prediction

## Abstract

**Aims:**

We aimed to explore the associations between urine albumin-to-creatinine ratio (uACR) and cardia-cerebrovascular disease (CVD) in Chinese population with type 2 diabetes(T2D).

**Methods:**

We included 8975 participants with T2D but free of prevalent CVD (including myocardial infarction, ischemic and hemorrhagic stroke) at baseline from Kailuan study who were assessed with uACR between 2014 and 2016. The participants were divided into three groups based on their baseline uACR: normal (< 3 mg/mmol), microalbuminuria (3–30 mg/mmol), and macroalbuminuria (≥ 30 mg/mmol). Cox regression models and restricted cubic spline were used to evaluate the hazard ratios (HRs) and 95% confidence intervals (CIs) of incident CVD. The area under the receiver operating characteristic curve (AUC), net reclassification improvement (NRI), and integrated discrimination improvement (IDI) were used to see if incorporating uACR into existing models could improve performance.

**Results:**

During a median follow-up of 4.05 years, 560 participants developed first CVD event (6.24%). After adjustment for potential confounders, participants with microalbuminuria had higher risks of CVD compared with normal uACR, with HRs of 1.57(95% CI 1.04–2.37) for myocardial infarction, 1.24(95% CI 1.00–1.54) for ischemic stroke,1.62(95% CI 0.73–3.61) for hemorrhagic stroke, and 1.30(95% CI 1.07–1.57) for total CVD. The risks gradually attenuated with uACR increase, with HRs of 2.86(95% CI 1.63–5.00) for myocardial infarction, 2.46(95% CI 1.83–3.30) for ischemic stroke, 4.69(95% CI 1.72–12.78) for hemorrhagic stroke, and 2.42(95% CI 1.85–3.15) for total CVD in macroalbuminuria. The addition of uACR to established CVD risk models improved the CVD risk prediction efficacy.

**Conclusions:**

Increasing uACR, even below the normal range, is an independent risk factor for new-onset CVD in T2D population. Furthermore, uACR could improve the risk prediction for CVD among community based T2D patients.

**Supplementary Information:**

The online version contains supplementary material available at 10.1186/s13098-024-01256-5.

## Introduction

China is a significant contributor to the burden of cardia-cerebrovascular diseases (CVD), with approximately 330 million individuals currently affected by CVD, including around 13 million stroke cases and 11.39 million cases of coronary heart disease [1]. The country is facing the dual challenges of an aging population and the persistent prevalence of metabolic risk factors, leading to a continuous increase in CVD prevalence and the highest mortality rate. As certain risk factors may be asymptomatic, patients may already have developed vasculopathy by the time these risk factors are detected, resulting in more severe events such as myocardial infarction or stroke. Diabetes is a major risk factor for CVD, and individuals with diabetes are considered a high-risk population for CVD. Currently, the prevalence of diabetes in China is 12.8% (according to ADA criteria), with a total of 129.8 million adults affected [2]. Therefore, the key focus of CVD prevention is to identify patients at high risk and implement appropriate interventions.

Diabetic microangiopathy may be one of the mechanisms of high risk of CVD. Albuminuria testing, specifically the measurement of urinary albumin-to-creatinine ratio (uACR), has been recommended to assess renal function in patients with type 2 diabetes(T2D) [3]. Albuminuria, an early-stage marker of blood vessel dysfunction and a sensitive indicator of diabetic microangiopathy, is increasingly recognized as a predictive indicator of cardiovascular risk and all-cause mortality in numerous studies [4]. Most studies have confirmed that elevated uACR is an independent risk factor for increased morbidity and mortality of CVD, whether in the general population [5–7], individuals at low risk of CVD (without diabetes or hypertension) [8, 9], or individuals with diabetes who have a high risk of CVD (8). Some research even suggests that this conclusion holds true when uACR is below the clinical threshold for albuminuria [10–12]. However, certain other studies have shown no significant difference between uACR and CVD in a cross-sectional analysis [13, 14].

The existing studies only examined the relationship between uACR and the overall occurrence of CVD events. However, no studies have explored the association between uACR and specific CVD events in the diabetic population. Therefore, in this study, we investigated the relationship between uACR in individuals with T2D and both the total number of CVD events and specific types of CVD events using data from the Kailuan study. Furthermore, we discussed the impact of incorporating uACR into the CVD prediction model.

## Methods

### Study populations

This prospective cohort study comprised in-service and retired Kailuan employees of the Kailuan Group, who participated in the physical examination conducted every two years in Kailuan General Hospital and the affiliated hospitals from June 2006 to October 2007. The follow-up included an evaluation of myocardial infarction, ischemic and hemorrhagic stroke. As urine albumin and creatinine tests were added during the physical examinations in 2014 (5th) and 2016 (6th), diabetic patients who underwent these tests and participated in the 5th and/or 6th physical examinations were enrolled.

Inclusion criteria: (1) those who participated in the 5th or 6th physical examination; (2) Participants who met the diagnostic criteria for type 2 diabetes; (3) those who had complete urine albumin and creatinine data, and (4) those who agreed for participation and signed informed consent. Exclusion criteria: (1) those with a previous history of CVD (including myocardial infarction, ischemic and hemorrhagic stroke); (2) those who did not agree to participate in this study. This study was conducted in accordance with the Declaration of Helsinki and was approved by the Ethics Committee of Kailuan General Hospital.

### Baseline information

Epidemiological investigations and biochemical and anthropological measurements were detailed in the published literature [15]. Subjects sat still for 15 min before measuring their blood pressure. A bench- top mercurial phygmomanometer was employed to measure the right brachial pressure. Three consecutive measurements were taken with an interval of 1–2 min between each measurement, and the average of the three measurements was considered. Smoking was defined as an average of ≥ 1 cigarette/day in the last year. Body mass index (BMI) = weight (kg)/height (m)^2^. The estimated glomerular filtration rate (eGFR) was calculated using the Chronic Kidney Disease Epidemiology Collaboration (CKD-EPI) equation [16].

### Urine albumin and urine creatinine determination and grouping

After an overnight fast, a single random midstream morning urine sample was collected. All participants’ morning urine samples were centrifuged at 600 g for 5 min and stored at − 80 °C until tested. A urine analyzer was used to measure all of the urine samples (N-600, Dirui, Changchun, China). Jaffe’s kinetic method was used to measure urinary creatinine. Turbidimetry was used to measure urinary albumin (DAKO kit, Denmark).

We looked at uACR as a continuous and categorical variable, with normal (uACR < 3 mg/mmol), microalbuminuria (3–30 mg/mmol), and macroalbuminuria (≥ 30 mg/mmol) categories [17, 18].

### Follow-up and end‐point event

After the completion of uACR determination, that is, the starting point of follow-up, trained medical staff reviewed the inpatient diagnosis and recorded the end‐point events of the observation objects in the Affiliated Hospitals of Kailuan Group and the Designated Hospitals for Medical and Health Insurance of China every year. The end‐point events were defined as CVD during the follow‐up, including myocardial infarction and ischemic and hemorrhagic stroke (please refer to the Standards from World Health Organization for their definitions and diagnostic criteria). Based on the inpatient medical records, professional doctors confirmed all diagnoses. The time and event of the first event were considered as the endpoint for those with ≥ 2 events, and the final follow‐up date for those without events was December 31, 2020.

### Statistical analysis

Normally distributed measurement data were expressed as mean + sd. Multiple pairwise-comparison between different groups was conducted using a one-way analysis of variance. The least significant difference (LSD) test and Dunnett’s T3 test were used for evaluating the homogeneity of variance and heterogeneity of variance, respectively. Non-normally distributed data were presented as median and centiles (25th and 75th), while the comparison between the groups was performed using the Kruskal-Wallis rank sum test. Enumeration data were presented as frequency and percentage (n, %), and comparisons between groups were performed by the chi-square test. The Kaplan-Meier method was used to calculate the incidence of CVD events in each group and the overall population, and a log-rank test was adopted to compare the difference in the incidence of CVD.

The uACR was assessed as a categorical and continuous variable. Given a non-normal distribution, uACR was ln-transformed for the continuous model. The effect of different uACR groups and each 1-standard deviation (SD) increase in ln (uACR) on new-onset CVD events was studied using a multivariate Cox stepwise regression model. Model 1 unadjusted. Model 2 was adjusted for age and gender. Model 3 was further adjusted for SBP, FBG, LDL cholesterol, BMI, eGFR, smoking, anti-diabetic treatment and antihypertensive treatment.

In addition, based on Model 2(age, gender), Model 4 (HKU-SG risk score: age, gender, SBP, DBP, HbA1c, LDL cholesterol, BMI, CKD (evaluated by eGFR), atrial fibrillation and smoking), the receiver operating characteristic (ROC) area under the curve (AUC), net reclassification index (NRI), and integrated discrimination improvement (IDI) were used to assess the ability of uACR to improve CVD prediction models, respectively.

A spline function curve was plotted to see if there was a linear correlation between uACR and new-onset CVD events. The multivariable adjusted model include age, gender, SBP, FBG, LDL cholesterol, BMI, eGFR, smoking, anti-diabetic treatment and antihypertensive treatment.

SAS version 9.4 was used for the analysis (SAS Institute, Cary, NC, USA). All statistical analyses were double-tailed, with statistical significance set at *P* < 0.05.

## Results

### Study cohort

A total of 1820 T2D patients participated in the 5th physical examination, which included urine albumin and creatinine tests, and 8827 in the 6th physical examination. However, 9642 patients were included in the study after excluding 167 and 500 patients who had incomplete urine albumin and creatinine data and a history of CVD events before the physical examination, respectively. Subsequently, 8975 patients were included in the statistical analysis(Supplementary Fig. S1).

### Cohort characteristics

The study population encompassed 6475 (72.14%) males and 2500 (27.86%) females, with a mean age of 61.10 ± 9.97 years and a mean SBP of 146.55 ± 20.62 mmHg, HbA1c of 7.59 ± 1.66%. The median value of uACR was 1.66 mg/mmol. Moreover, 66.14% of the overall population had uACR in the normal range, and 27.96% and 5.90% of them had microalbuminuria and macroalbuminuria, respectively.

Table 1 shows the demographic and clinical characteristics of the study population. Compared to those in the normal uACR group, the patients with microalbuminuria and macroalbuminuria had higher levels of SBP, DBP, FBG, HbA1c, total cholesterol, LDL-cholesterol, triglycerides, BMI, heart rate and hypertension prevalence and CVD prevalence as well as a lower eGFR level (Table 1).

**Table 1:** Baseline Characteristics Overall and by uACR Categories in Participants

	overall (*n* = 8975)	< 3 mg/mmol (*n* = 5936)	3-30 mg/mmol (*n* = 2509)	≥ 30 mg/mmol (*n* = 530)	*P*-value
Myocardial infarction, *n* (%)	118(1.31)	56(0.94)	43(1.71)	19(3.58)	< 0.001
Ischemic stroke, *n* (%)	436(4.86)	227(3.82)	145(5.78)	64(12.08)	< 0.001
Hemorrhagic stroke, *n* (%)	33(0.37)	16(0.27)	11(0.44)	6(1.13)	0.006
Total CVD events, *n* (%)	560(6.24)	288(4.85)	191(7.61)	81(15.28)	< 0.001
Male, *n* (%)	6475(72.14)	4295(72.36)	1792(71.42)	388(73.21)	0.583
Age, years	61.03 ± 9.98	60.34 ± 9.84	62.08 ± 10.14	63.70 ± 9.98	0.302
uACR*, mg/mmol	1.66 (0.80 4.55)	1.01 (0.63 ~ 1.64)	6.16 (4.13 ~ 11.28)	61.32 (42.46 ~ 111.37)	< 0.001
SBP, mmHg	146.55 ± 20.62	143.49 ± 19.61	151.63 ± 20.88	156.85 ± 22.38	< 0.001
DBP, mmHg	82.83 ± 10.97	81.83 ± 10.40	84.50 ± 11.65	86.12 ± 12.17	< 0.001
Heart rate, bpm	77.453 ± 12.89	76.63 ± 12.55	79.10 ± 13.31	80.09 ± 13.42	0.006
BMI, kg/mm^2^	25.79 ± 3.44	25.58 ± 3.32	26.14 ± 3.59	26.49 ± 3.724	0.002
Triglycerides *, mmol/L	153 (1.05 ~ 2.32)	1.45 (1.00 ~ 2.18)	1.69 (1.15 ~ 2.63)	1.84 (1.24 ~ 2.80)	0.018
Total cholesterol, mmol/L	5.49 ± 1.18	5.42 ± 1.13	5.59 ± 1.22	5.79 ± 1.41	< 0.001
HDL-C*, mmol/L	1.38 (1.18 ~ 1.62)	1.39(1.19 ~ 1.65)	1.34 (1.16 ~ 1.58)	1.32 (1.12 ~ 1.58)	0.635
LDL-C, mmol/L	3.24 ± 0.95	3.21 ± 0.93	3.30 ± 0.99	3.40 ± 1.07	0.001
FBG, mmol/L	9.10 ± 3.26	8.55 ± 2.94	10.07 ± 3.50	10.76 ± 3.84	< 0.001
HbA1c, %	7.59 ± 1.66	7.32 ± 1.53	8.07 ± 1.75	8.41 ± 1.84	< 0.001
Hemoglobin, g/L	150.49 ± 14.53	150.57 ± 14.11	150.73 ± 14.69	148.35 ± 17.86	< 0.001
hsCRP *	1.10 (0.34 ~ 2.77)	0.92 (0.29 ~ 2.41)	1.43 (0.49 ~ 3.42)	1.77 (0.73 ~ 3.80)	0.566
eGFR, ml/min/1.73m^2^	82.81 ± 19.61	83.54 ± 18.95	82.69 ± 19.58	75.22 ± 24.77	< 0.001
Smoking, *n* (%)	3018(33.63)	2026(34.13)	831(33.12)	161(30.38)	0.176
Hypertension, *n* (%)	5071(56.50)	3057(51.50)	1644(65.52)	370(69.81)	< 0.001
Atrial fibrillation, *n* (%)	94(1.05)	55(0.93)	33(1.32)	6(1.13)	0.271
Anti-diabetic treatment, *n* (%)	3749(41.77)	2222(37.43)	1209(48.19)	318(60.00)	< 0.001
Insulin,	1581(17.62)	877(14.77)	527(21.00)	177(33.40)	< 0.001
Oral medicine	2173(24.21)	1348(22.71)	683(27.22)	142(26.79)	< 0.001
Antihypertensive treatment, *n* (%)	3754(41.83)	2382(40.13)	1087(43.32)	285(53.77)	< 0.001

CVD: cardia-cerebrovascular disease; uACR: urine albumin-to-creatinine ratio; SBP: systolic blood pressure; DBP: diastolic blood pressure; BMI: body mass index; HDL-C: high density lipoprotein-cholesterol; LDL-C: low density lipoprotein-cholesterol; FBG: fasting blood glucose; hs-CRP: highly sensitive C-reactive protein; eGFR: estimated glomerular filtration rate; * expressed in M(Q1 ~ Q3)

### Cumulative incidence of CVD events in each uACR groups

**Fig. 1:**
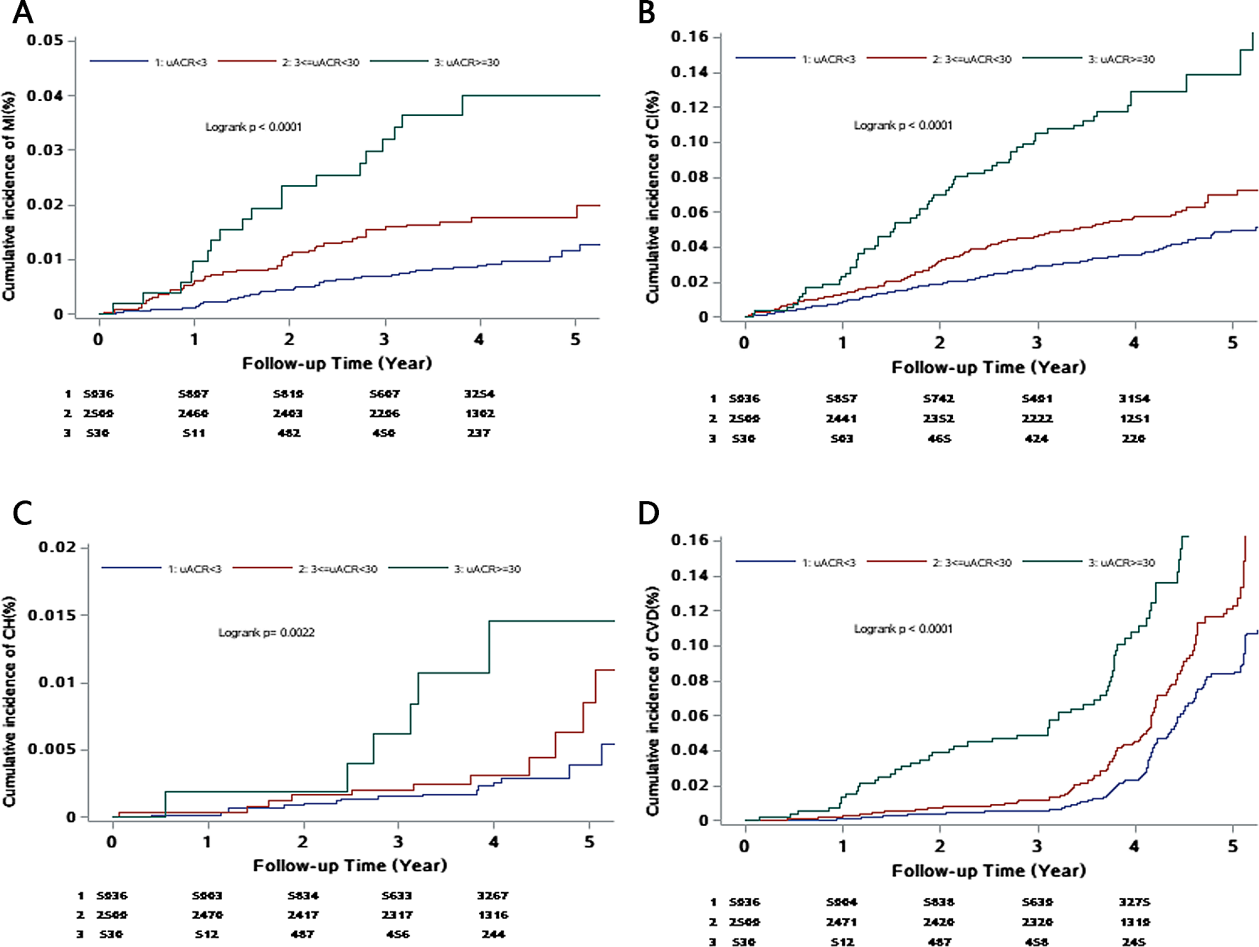
Cumulative incidence of CVD events in each uACR groups (N = 8975). A: Incidence of myocardial infarction by uACR category; B: Incidence of ischemic stroke by uACR category; C: Incidence of hemorrhagic stroke by uACR category; D: Incidence of total CVD events by albuminuria category: albuminuria categories were based on urinary albumin-creatinine ratios(uACR) as macroalbuminuria(uACR ≥ 30 mg/mmol), microalbuminuria(uACR < 30 to ≥ 3 mg/mmol), and normal(uACR < 3 mg/mmol).

During the median 4.05(3.55, 4.48) years of follow-up, 118 participants (1.31%) developed myocardial infarction, 436 participants (4.86%) and 33 participants (0.37%) developed ischemic and hemorrhagic stroke, respectively. In total, 560 cases of CVD events were reported, with cumulative incidence of 1.55%, 3.37%, and 15.07% in all three groups, respectively. A log-rank test showed a significant difference in the cumulative incidence between the groups (Fig. 1).

### Cox proportional-hazards model affecting CVD events

Considering CVD events as the dependent variable and uACR groups or per 1-SD increase in ln (uACR) as the independent variables, and the normal uACR as the reference group, age, gender, SBP, FBG, LDL-cholesterol, BMI, eGFR, smoking, anti-diabetic treatment and antihypertensive treatment were corrected. Cox regression analysis confirmed that the adjusted risks of myocardial infarction in patients with microalbuminuria and macroalbuminuria were 1.57(95% CI 1.04 ~ 2.37) and 2.86 (95% CI 1.63 ~ 5.00), respectively; the adjusted risks of ischemic stroke in two groups were 1.24(95% CI 1.00 ~ 1.54) and 2.46 (95% CI 1.83 ~ 3.30), respectively; the adjusted risks of hemorrhagic stroke in two groups were 1.62(95% CI 0.73 ~ 3.61) and 4.69 (95% CI 1.72 ~ 12.78), respectively; the adjusted risks of total CVD events in two groups were 1.30(95% CI 1.07 ~ 1.57) and 2.42 (95% CI 1.85 ~ 3.15), respectively (Table 2).

**Table 2:** Hazard ratios(HRs) and 95%Confidence intervals of uACR for CVD events

	No.	Events	No./1000 person-years	Model 1	Model 2	Model 3
Myocardial infarction						
< 3 mg/mmol	5936	56	2.32	1	1	1
3-30 mg/mmol	2509	43	4.3	1.86(1.25, 2.76)	1.71(1.15, 2.56)	1.57(1.04, 2.37)
≥ 30 mg/mmol	530	19	9.53	4.10(2.44, 6.90)	3.58(2.12, 6.05)	2.86(1.63, 5.00)
ln(uACR),Per 1 SD	8975	118	3.26	1.64(1.42, 1.90)	1.58(1.34, 1.84)	1.80(1.24, 2.61)
Ischemic stroke						
< 3 mg/mmol	5936	227	9.53	1	1	1
3-30 mg/mmol	2509	145	14.83	1.55(1.26, 1.91)	1.46(1.19, 1.80)	1.24(1.00, 1.54)
≥ 30 mg/mmol	530	64	33.33	3.50(2.65, 4.62)	3.11(2.36, 4.12)	2.46(1.83, 3.30)
ln(uACR),Per 1 SD	8975	436	12.28	1.49(1.38, 1.62)	1.44(1.33, 1.57)	1.34(1.22, 1.46)
Hemorrhagic stroke						
< 3 mg/mmol	5936	16	0.66	1	1	1
3-30 mg/mmol	2509	11	1.09	1.66(0.77, 3.58)	1.55(0.72, 3.36)	1.62(0.73, 3.61)
≥ 30 mg/mmol	530	6	2.97	4.69(1.84, 11.99)	4.16(1.62, 10.72)	4.69(1.72, 12.78)
ln(uACR),Per 1 SD	8975	33	0.91	1.51(1.13, 2.01)	1.45(1.08, 1.96)	1.10(0.55, 2.19)
Total CVD events						
3-30 mg/mmol	5936	288	11.87	1	1	1
3-30 mg/mmol	2509	191	18.97	1.60(1.33, 1.92)	1.45(1.20, 1.74)	1.30(1.07, 1.57)
≥ 30 mg/mmol	530	81	40.16	3.57(2.79, 4.572)	2.94(2.29, 3.77)	2.42(1.85, 3.15)
ln(uACR),Per 1 SD	8975	560	15.41	1.52(1.42, 1.63)	1.44(1.34, 1.55)	1.37(1.26, 1.48)

Model 1: unadjusted;

Model 2: adjusted for age and sex;

Model 3: adjusted for age, gender, SBP, FBG, LDL-cholesterol, BMI, eGFR, smoking, anti-diabetic treatment and antihypertensive treatment

### Restrictive cubic spline multivariable cox regression analysis was used to analyze the relationship between uACR and the risk of new-onset CVD events

**Fig. 2:**
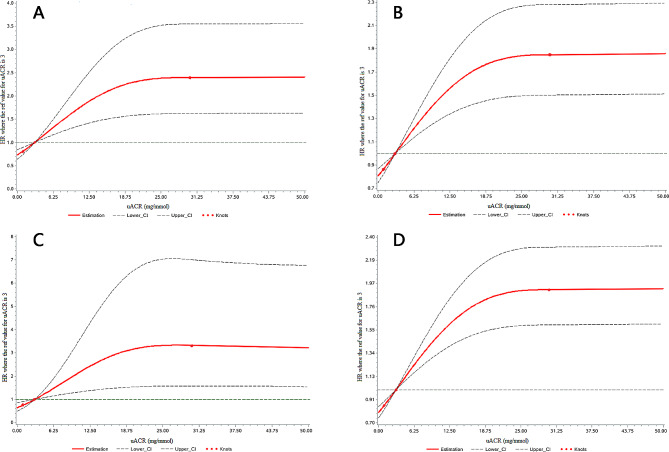
Association between CVD events and the continuous level of urinary albumin-to-creatinine ratios(uACR) using RCS with 3 knots (in 8975 participants): (A) Myocardial infarction; (B) Ischemic stroke; (C) Hemorrhagic stroke; (D) Total CVD events. The reference point is uACR of 3 mg/mmol. The solid lines represent the hazard ratios across the spectrum of uACR. The dashed lines represent the upper and lower bounds of the 95% confidence interval. P-values reflect adjusted trends (accounting for age, gender, SBP, FBG, LDL-cholesterol, BMI, eGFR, smoking, anti-diabetic treatment and antihypertensive treatment)

The overall and nonlinear associations between uACR and new-onset CVD were statistically significant (*p* < 0.001). The results of the restrictive cubic spline multivariable Cox regression analysis indicated that the risks of CVD events gradually increased with an increase in uACR after adjustment for covariates (Fig. 2).

### Additional predictive value of uACR for established CVD risk model

In order to explore the incremental predictive value of uACR for CVD events, we evaluated the effect of adding uACR to Model 2 (including age and sex) and Model 4 (the HKU-SG risk score: age, sex, SBP, DBP, HbA1c, LDL cholesterol, BMI, CKD(eGFR), atrial fibrillation and smoking) [19], respectively. As shown in Table 3, the addition of uACR to the known models improved the predictivity of CVD risk (*p* < 0.001).

**Table 3:** The additional predictive value of uACR for CVD events

		AUC	NRI	*P*-value	IDI	*P*-value
Myocardial infarction	Model 2	0.641(0.594, 0.689)	ref	-	ref	-
	Model 2 + ln(uACR)	0.679(0.628, 0.730)	0.213(0.121, 0.304)	< 0.001	0.006(0.002, 0.013)	< 0.001
	Model 2 + uACR categories	0.668(0.618, 0.718)	0.226(0.131, 0.315)	< 0.001	0.004(0.001, 0.009)	< 0.001
	Model 4*	0.684(0.635, 0.733)	ref	-	ref	-
	Model 4 + ln(uACR)	0.701(0.653,0.749)	0.139(0.041, 0.240)	< 0.001	0.005(0.002, 0.013)	< 0.001
	Model 4 + uACR categories	0.693(0.645, 0.741)	0.211(0.095, 0.301)	< 0.001	0.003(0.001, 0.009)	< 0.001
Ischemic stroke	Model 2	0.599(0.574, 0.625)	ref	-	ref	-
	Model 2 + ln(uACR)	0.645(0.619, 0.671)	0.161(0.101, 0.216)	< 0.001	0.012(0.006, 0.019)	< 0.001
	Model 2 + uACR categories	0.638(0.612, 0.664)	0.176(0.125, 0.218)	< 0.001	0.008(0.004, 0.015)	< 0.001
	Model 4*	0.665(0.639, 0.689)	ref	-	ref	-
	Model 4 + ln(uACR)	0.676(0.651, 0.701)	0.121(0.067, 0.172)	< 0.001	0.007(0.003, 0.014)	< 0.001
	Model 4 + uACR categories	0.672(0.647, 0.698)	0.167(0.116, 0.211)	< 0.001	0.005(0.002, 0.010)	< 0.001
Hemorrhagic stroke	Model 2	0.588(0.485, 0.692)	ref	-	ref	-
	Model 2 + ln(uACR)	0.644(0.538, 0.750)	0.178(-0.112, 0.397)	0.229	0.001(0.000, 0.007)	0.05
	Model 2 + uACR categories	0.649(0.550, 0.749)	0.165(-0.044, 0.365)	0.09	0.001(0.000, 0.008)	0.030
	Model 4*	0.666(0.569, 0.762)	ref	-	ref	-
	Model 4 + ln(uACR)	0.703(0.600, 0.806)	0.164(-0.102, 0.365)	0.269	0.001(0.000, 0.017)	0.109
	Model 4 + uACR categories	0.712(0.610, 0.813)	0.163(-0.058, 0.379)	0.119	0.001(0.000, 0.016)	0.139
Total CVD events	Model 2	0.604(0.582, 0.627)	ref	-	ref	-
	Model 2 + ln(uACR)	0.653(0.630, 0.676)	0.191(0.111, 0.256)	< 0.001	0.011(0.006, 0.018)	< 0.001
	Model 2 + uACR categories	0.644(0.621, 0.666)	0.221(0.152, 0.285)	< 0.001	0.008(0.004, 0.013)	< 0.001
	Model 4*	0.660(0.638, 0.682)	ref	-	ref	-
	Model 4 + ln(uACR)	0.678(0.656, 0.700)	0.121(0.053, 0.193)	< 0.001	0.008(0.004, 0.014)	< 0.001
	Model 4 + uACR categories	0.674(0.652, 0.695)	0.199(0.116, 0.261)	< 0.001	0.005(0.002, 0.011)	< 0.001

Model 2: age, sex; Model 4 (HKU-SG risk score): age, gender, SBP, DBP, HbA1c, LDL cholesterol, BMI, CKD(eGFR), atrial fibrillation and smoking

Abbreviation: uACR, urine albumin-to-creatinine ratio; CVD: cardiovascular and cerebrovascular disease; AUC: the area under the receiver operating characteristic curve; NRI: net reclassification improvement; IDI: integrated discrimination improvement

### Association between uACR and risk for CVD events in stratified analyses

We further analyzed the association between albuminuria evaluated by uACR and risk for CVD events in subgroups of sex, eGFR (≥ 60 ml/min/1.73m^2^ vs. < 60 ml/min/1.73m^2^), the presence of hypertension, and BMI (≥ 28 kg/mm^2^ vs. < 28 kg/mm^2^) (Supplementary Table S1).

The HRs for high total CVD risk significantly increased from the microalbuminuria in BMI < 28 kg/mm^2^, hypertensive participants, eGFR ≥ 60 ml/min/1.73m^2^, and in females, whereas in obese, nonhypertensive participants, eGFR < 60 ml/min/1.73m^2^ and in males, the HRs rose markedly from the macroalbuminuria.

## Discussion

This study indicates that in patients with T2D, an increase in uACR is an independent risk factor for myocardial infarction, ischemic and hemorrhagic stroke, as well as total CVD events. The relationship between uACR and total CVD events remains consistent across different populations, including those with varying genders, BMI, eGFR, and the presence of hypertension. Moreover, there is a dose-response relationship between uACR levels and the incidence risk of CVD events. Furthermore, incorporating uACR into established CVD risk prediction models improves the accuracy of predicting CVD risk.

Previous research have confirmed the relationship between uACR and CVD in the general population and in people with diabetes [12, 20], but above researches focused on total CVD events as endpoints, yielding inconsistent results. Results from the HOPE study [12] indicated that for every 0.4 mg/mmol increase in uACR, the risk of CVD increased by 5.9% after adjusting for age and sex in individuals with or without diabetes. The SHS [10] observed among all partticipants, risk of developing all CVD events increased by 13% for every doubling of uACR within the normal range, but among participants with diabetes, risk of all CVD events increased by 20%. A meta-analysis [6] confirmed that high uACR was associated with increased risk of ischemic stroke (HR, 1.60; 95% CI: 1.43–1.80), as well as hemorrhagic stroke (HR, 1.76; 95% CI: 1.22–1.45). Whereas subgroup analysis revealed high uACR was unable to predict stroke in patients with T2DM (HR, 2.25; 95% CI: 0.55–9.17). Aguilar [7] indicated among community-dwelling older adults, uACR was strongly associated with risk of incident stoke of any type and ischemic strok, but not hemorrhagic stroke. However, this study not only confirms the independent association between increased uACR and long-term incident total CVD events in patients with T2D but also examines the relationship between uACR and different types of CVD events. Additionally, our previous research has already demonstrated the link between increased uACR and an elevated long-term risk of heart failure in patients with T2D [21].

Specifically, this study reveals that in T2D patients with microalbuminuria (uACR levels ranging from 3 to 30 mg/mmol), the incidence risk of developing myocardial infarction, cerebral infarction, and total CVD events increases by 2.86-fold (95% CI: 1.04–2.37), 2.46-fold (95% CI: 1.00–1.54), and 1.30-fold (95% CI: 1.07–1.57), respectively. For patients with macroalbuminuria, the risk escalates by 2.86-fold (95% CI: 1.63–5.00), 2.46-fold (95% CI: 1.83–3.30), and 2.42-fold (95% CI: 2.42–3.15), respectively. Notably, when compared to individuals with normal uACR levels, those with macroalbuminuria have a significantly increased risk of hemorrhagic stroke by 4.69-fold (95% CI: 1.72–12.78), while the risk is not significantly different for individuals with microalbuminuria.

Our findings demonstrate that an increase in uACR is not only an independent risk factor for the development of CVD events in patients with T2D but also shows a dose-response relationship with the risk of CVD. Even when the level of albuminuria below the clinical threshold value of 3 mg/mmol, for the patients with T2D, an increase in uACR is associated with a significant elevation in CVD risk, which is consistent with previous studies [8, 11, 22]. Additionally, we observed that as uACR increased to a certain level, the growth trend of the risk for each specific CVD event and total CVD events became less steep than before. Similar findings were reported in the ARIC study [13], where an increased risk of heart failure was observed at a relatively high normal uACR level (approximately 1–3 mg/mol), with a relatively slower growth trend. These studies suggest that for the prevention of long-term CVD events in diabetes patients, a lower uACR level may be preferable.

In recent years, numerous epidemiological surveys and clinical studies on CVD risk factors have indicated that, in addition to traditional factors such as age, coronary heart disease, hypertension, and hyperglycemia, several other factors require further investigation and validation due to their close association with CVD incidence. The PREVEND study [23] showed that albuminuria, measured in 24-hour urine samples, is associated independently with cardiovascular outcomes (including myocardial infarction and stroke) in the general population and adding albuminuria to a model consisting of Framingham risk factors significantly contributed to identifying individuals at risk of cardiovascular outcomes. Currently, urinary albumin testing(spot urine sample) is already recommended for all patients with T2D to assess for chronic kidney disease. uACR could therefore readily be used as a more formal tool for cardiovascular risk prognostication. Our study confirms that incorporating uACR into established CVD risk prediction models, specifically by integrating uACR into the HKU-SG risk scoring, can enhance the accuracy of predicting the risk of developing CVD. The SAVOR-TIMI53 Trial [24] found that in patients with T2D, uACR was independently associated with increased risk for cardiovascular outcomes (cardiovascular death, myocardial infarction, or ischemic stroke), and uACR offers incremental prognositic benefit when cardiac biomarker was not considered. These results suggest that uACR can provide a predictive value beyond the traditional risk factors for CVD in patients with T2D, so uACR should be monitored regularly in the early stages of diabetes.

The underlying mechanisms underlying the associations between albuminuria and CVD risk are not well established. However, abnormal albuminuria indicates generalized vascular dysfunction and is related to systemic and coronary atherosclerosis [25, 26]. In patients with T2D, increased uACR are likely an early signal of microvascular disease and indicate some degree of kidney damage [27].the widespread vascular disorder may progress to loss of vessel distension and generalized elevation of arterial blood pressure, ultimately predispose patients to the development of micro- and macrovascular disease [11]. Consistent with previous studies, we also found that the effect of uACR on CVD events was independent of eGFR, these findings may support the hypothesis that albuminuria conferring risk of incident CVD is independent of renal filtration function [7]. Moreover, common risk factors may underlie the association between albuminuria and CVD events [28].However, even after full adjustment of conventional cardiovascular risk factors and other potential confounders, albuminuria was still significantly associated with high CVD risk in the present study, suggesting that independent and additional mechanisms may be involved. Also, the association between albuminuria and CVD events is probably explained by a common pathophysiologic process, such as endothelial dysfunction [29] or chronic, low-grade inflammation [30]. Further studies are clearly required to expand our understanding in this field.

Prominent strengths of this study are its prospective design, large sample size, multivariable-adjusted analyses and continuous surveillance and careful confirmation of incident CVD events. However, our study also has several limitations. First, uACR was only measured once at baseline in this study; thus, we cannot exclude intrapatient sampling variability. Secondly, the identification of myocardial infarction and stroke cases was based on hospitalization codes, but we did not include clinical data related to specific symptoms of CVD, which may have excluded patients who did not receive hospital treatment and did not provide a more holistic understanding of the study population’s cardiovascular health. Moreover, the duration of follow-up in our study was relatively short, with a median follow-up time of 4.05 years, and therefore, some endpoint events may not have fully occurred. Also, the number of events for hemorrhagic stroke was small, resulting in compromised ability to adjust for covariates and wide Cis and thus needing caution when interpreting relevant results. Finally, because the study participants were mostly male Kailuan Group employees, the extrapolation of results may be limited. However, the results in the male and female populations were both consistent with those in the overall population after gender subgrouping.

## Conclusion

Among community T2D patients free of prior CVD events, albuminuria reflected by uACR was significantly and independently associated with risk of incident CVD of any type and myocardial infarction, ischemic stroke and hemorrhagic stroke, independent of known risk determinants. As such, albuminuria, even below the traditional thresholds for defining microalbuminuria, is potentially useful for improved cerebro-cardiovascular risk stratification in patients with T2D.

### Electronic supplementary material

Below is the link to the electronic supplementary material.

Supplementary Material 1: Figure S1. Flowchart of the current study. Table S1. Hazard ratios (HR) and 95% Confidence intervals of uACR for CVD (subgroup analysis according to sex, eGFR, BMI and Hypertension).

## Data Availability

The datasets used and/or analyzed during the current study are available from the corresponding author on reasonable request.

## Abbreviations

uACR: Urine albumin-to-urine creatinine ratio
CVD: Cardia-cerebrovascular disease
T2D: Type 2 diabetes
HRs: Hazard ratios
CIs: Confidence intervals
AUC: Area under the receiver operating characteristic curve
NRI: Net reclassification improvement
IDI: Integrated discrimination improvement
BMI: Body mass index
eGFR: Estimated glomerular filtration rate
CKD-EPI: Chronic Kidney Disease Epidemiology Collaboration
SD: Standard deviation
FBG: Fasting blood glucose
HbA1c: Hemoglobin A1c
SBP: Systolic blood pressure
DBP: Diastolic blood pressure
LDL: Low-density lipoprotein
HDL: High-density lipoprotein
hs-CRP: High sensitivity C-reactive protein
The HKU-SG risk score: Age, sex, SBP, DBP, HbA1c, LDL cholesterol, BMI, CKD(eGFR), atrial fibrillation and smoking
