# Supplementary material for: An elevated urinary albumin-to-creatinine ratio increases the risk of incident cardia-cerebrovascular disease in individuals with type 2 diabetes
Source: Diabetol Metab Syndr. 2024 Jan 31;16:30. doi: 10.1186/s13098-024-01256-5 (PMC10829292; doi:10.1186/s13098-024-01256-5)
Supplement: Supplementary file 1 — Supplementary Material 1: Figure S1. Flowchart of the current study. Table S1. Hazard ratios (HR) and 95% Confidence intervals of uACR for CVD (subgroup analysis according to sex, eGFR, BMI and Hypertension). [file 13098_2024_1256_MOESM1_ESM.docx]

**Supplemental Material**

**Supplementary Figure S1.** Flowchart of the current study.


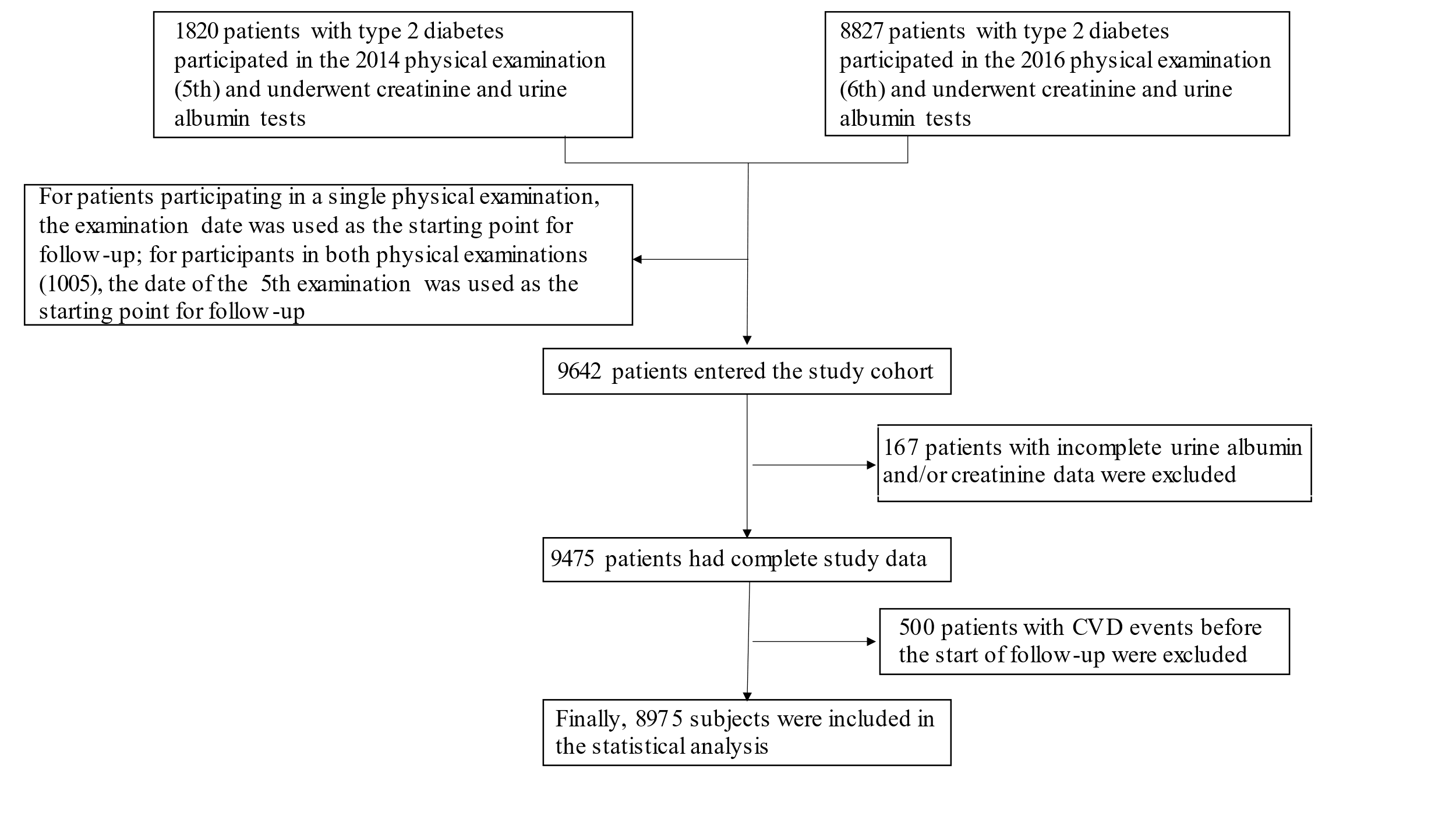
Fig. 1 Flowchart of the current study.

**Supplementary Table S1.** Hazard ratios (HR) and 95% Confidence intervals of uACR for CVD (subgroup analysis according to sex, eGFR, BMI and Hypertension)

**Table 1** Association between uACR and risk for CVD events in stratified analyses

|  | Myocardial infarction | | | | Ischemic stroke | | | |
| --- | --- | --- | --- | --- | --- | --- | --- | --- |
|  | Event/total | Incidence Rate  (/1000 person-years) | 3-30mg/mmol | ≥30 mg/mmol | Event/total | Incidence Rate  (/1000 person-years) | 3-30mg/mmol | ≥30 mg/mmol |
| Sex | | | | | | | | |
| Males | 80/6475 | 3.11 | 1.67(1.02, 2.82) | 3.33(1.73, 6.41) | 315/6475 | 11.79 | 1.17(0.90, 1.52) | 2.50(1.77, 3.53) |
| Females | 38/2500 | 3.64 | 1.31(0.64, 2.68) | 1.75(0.57, 5.38) | 121/2500 | 12.47 | 1.42(0.95, 2.12) | 2.36(1.32, 4.24) |
| eGFR | | | | | | | | |
| ≥60 | 96/7909 | 2.97 | 1.43(0.91, 2.23) | 1.79(0.86, 3.75) | 373/7909 | 11.74 | 1.30(1.03, 163) | 2.55(1.83, 3.55) |
| <60 | 22/1066 | 5.76 | 3.19(0.93, 10.99) | 8.06(2.23, 29.19) | 63/1066 | 16.86 | 0.94(0.51, 1.73) | 1.88(0.96, 3.69) |
| Hypertension | | | | | | | | |
| Yes | 72/5071 | 3.53 | 1.60(0.94, 2.72) | 3.06(1.54, 6.04) | 288/5071 | 14.42 | 1.20(0.92, 1.55) | 2.10(1.47, 2.99) |
| No | 46/3904 | 2.92 | 1.41(0.72, 2.78) | 2.41(0.88, 6.61) | 148/3904 | 9.52 | 1.35(0.92, 1.99) | 3.40(2.00, 5.79) |
| BMI | | | | | | | | |
| <28 | 96/6971 | 3.41 | 1.44(0.92, 2.28) | 2.25(1.15, 4.38) | 339/3857 | 12.27 | 1.20(0.94, 1.54) | 2.65(1.90, 3.70) |
| ≥28 | 22/2004 | 2.74 | 2.66(0.93, 7.65) | 6.70(2.06, 21.81) | 97/5430 | 12.31 | 1.34(0.86, 2.09) | 1.92(1.02, 3.62) |
|  | Hemorrhagic stroke | | | | Total CVD events | | | |
|  | Event/total | Incidence Rate  (/1000 person-years) | 3-30mg/mmol | ≥30 mg/mmol | Event/total | Incidence Rate  (/1000 person-years) | 3-30mg/mmol | ≥30 mg/mmol |
| Sex | | | | | | | | |
| Males | 22/6475 | 0.85 | 1.69(0.65, 4.39) | 3.07(0.79, 12.02) | 396/6475 | 15.33 | 1.22(0.97, 1.54) | 2.40(1.75, 3.29) |
| Females | 11/2500 | 1.05 | 1.36(0.31, 6.02) | 7.96(1.75, 36.33) | 164/2500 | 15.61 | 1.46(1.04, 2.05) | 2.21(1.32, 3.68) |
| eGFR | | | | | | | | |
| ≥60 | 28/7909 | 0.86 | 1.41(0.60, 3.32) | 3.69(1.15, 11.82) | 475/7909 | 14.62 | 1.34(1.10, 1.64) | 2.38(1.75, 3.22) |
| <60 | 5/1066 | 1.30 | 4.34(0.37, 49.99) | 0.13(0.73,140.76) | 85/1066 | 22.14 | 1.11(0.65, 1.90) | 2.57(1.43, 4.62) |
| Hypertension | | | | | | | | |
| Yes | 21/5071 | 1.02 | 1.60(0.58, 4.42) | 5.59(1.71, 18.28) | 362/5071 | 17.64 | 1.29(1.03, 1.63) | 2.19(1.59, 3.03) |
| No | 12/3904 | 0.76 | 1.78(0.49, 6.48) | 3.04(0.35, 26.17) | 198/3904 | 12.52 | 1.28(0.92, 1.80) | 3.06(1.92, 4.88) |
| BMI | | | | | | | | |
| <28 | 28/6971 | 0.99 | 1.76(0.24, 12.83) | 2.76(0.22, 34.18) | 441/6971 | 15.60 | 1.28(1.03, 1.59) | 2.53(1.88, 3.43) |
| ≥28 | 5/2004 | 0.62 | 1.78(1.21, 2.62) | 4.47(2.87, 6.97) | 119/2004 | 14.74 | 1.28(0.85, 1.92) | 2.10(1.20, 3.67) |

Adjust for age, sex, SBP,FBG, LDL-C, BMI, eGFR, smoking, Anti-diabetic treatment and antihypertensive treatment.
